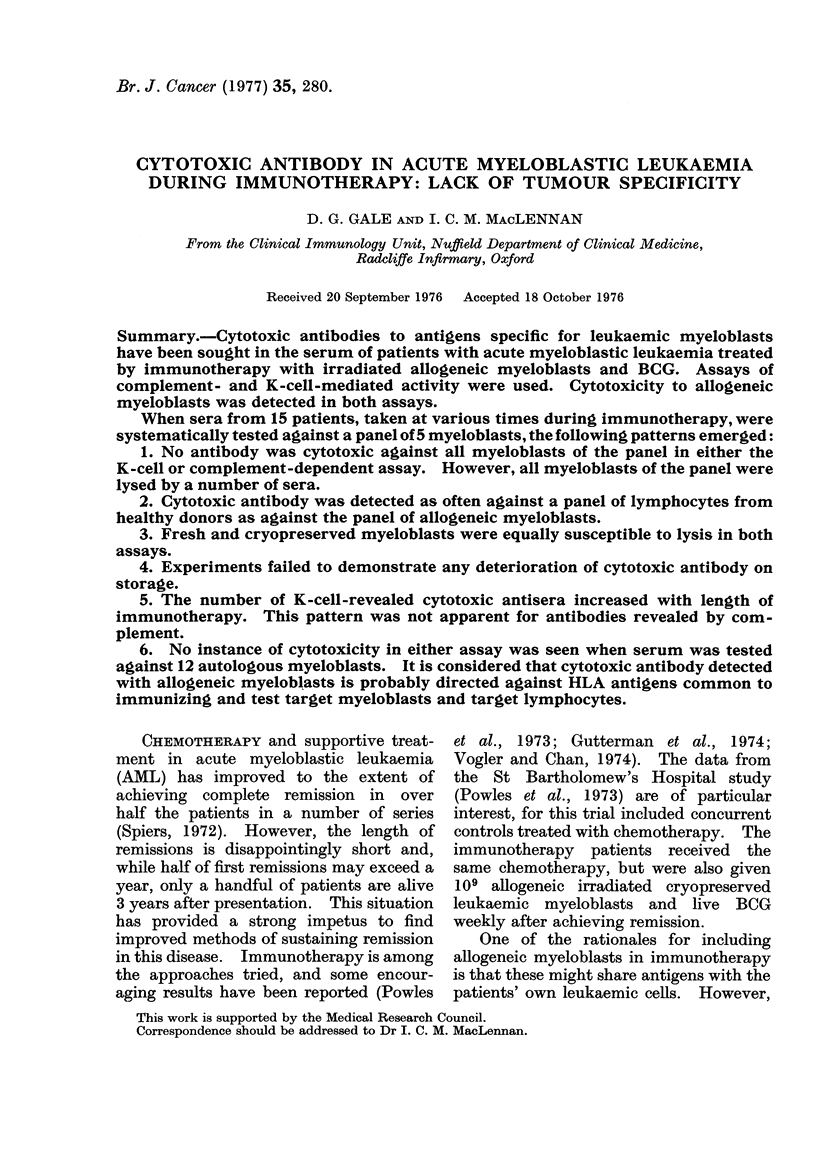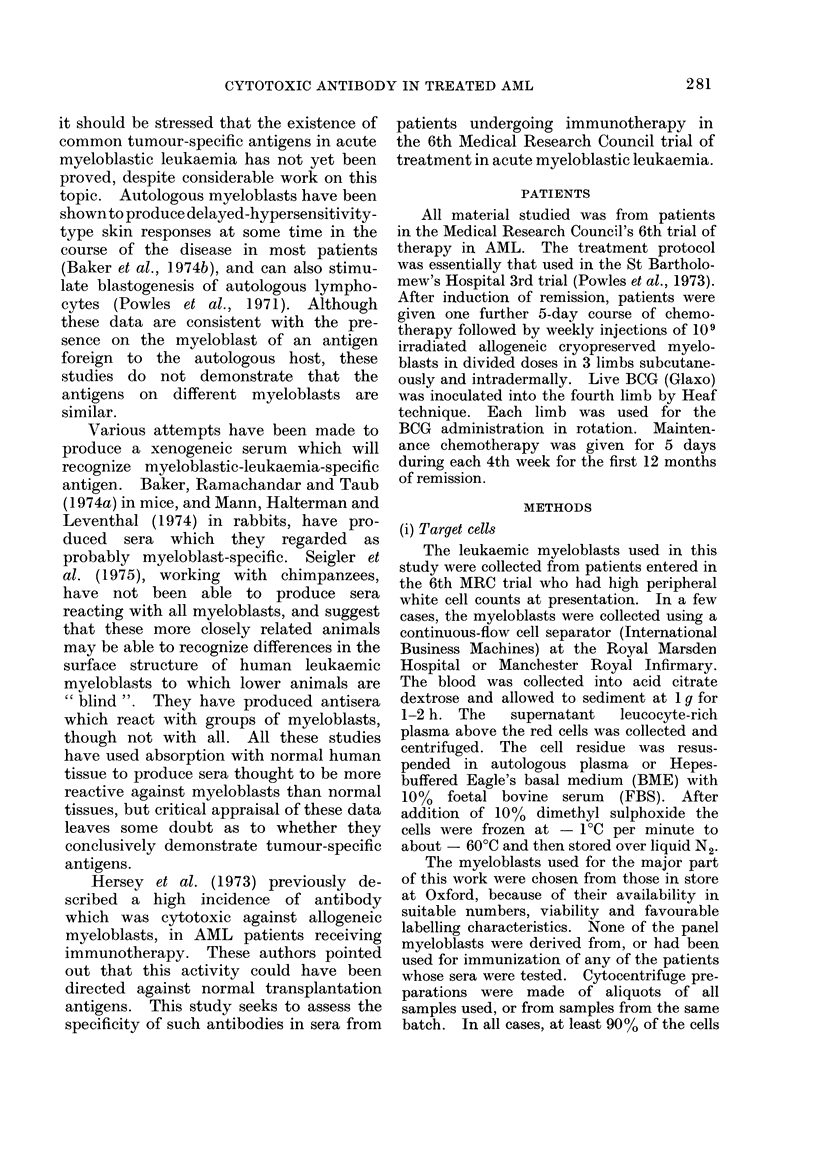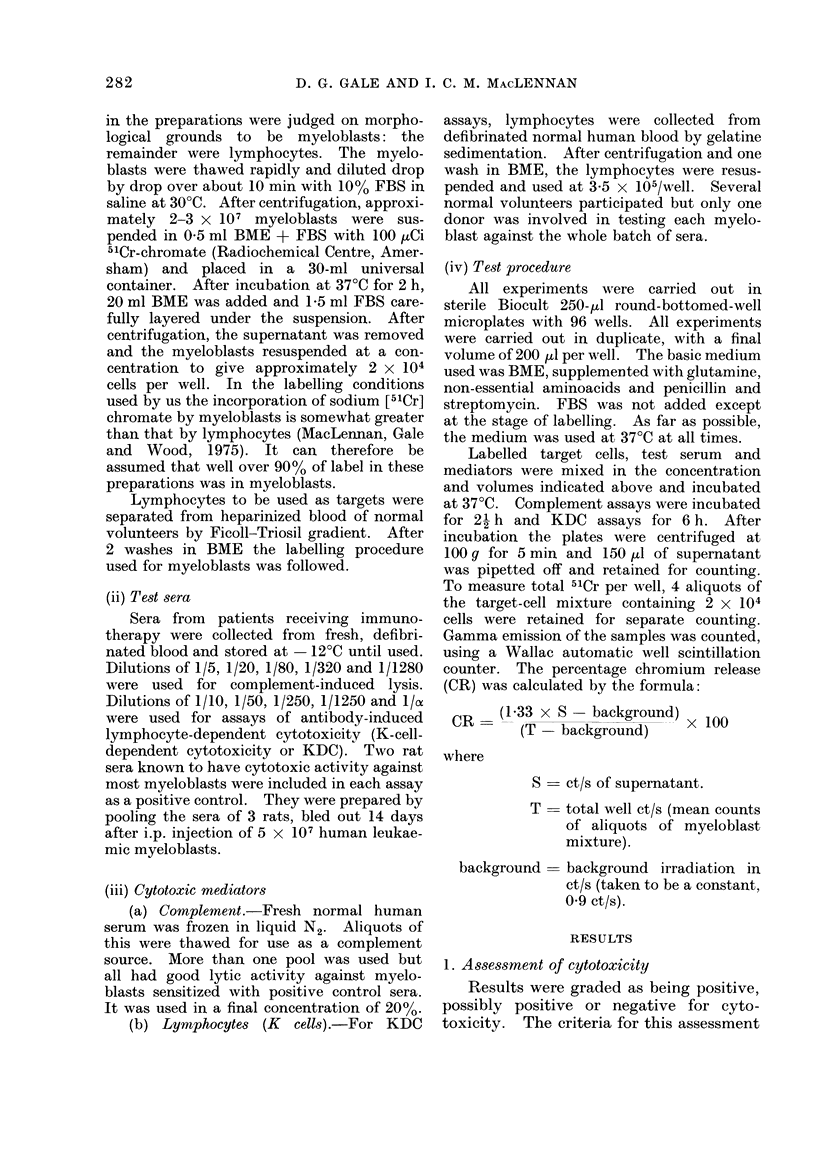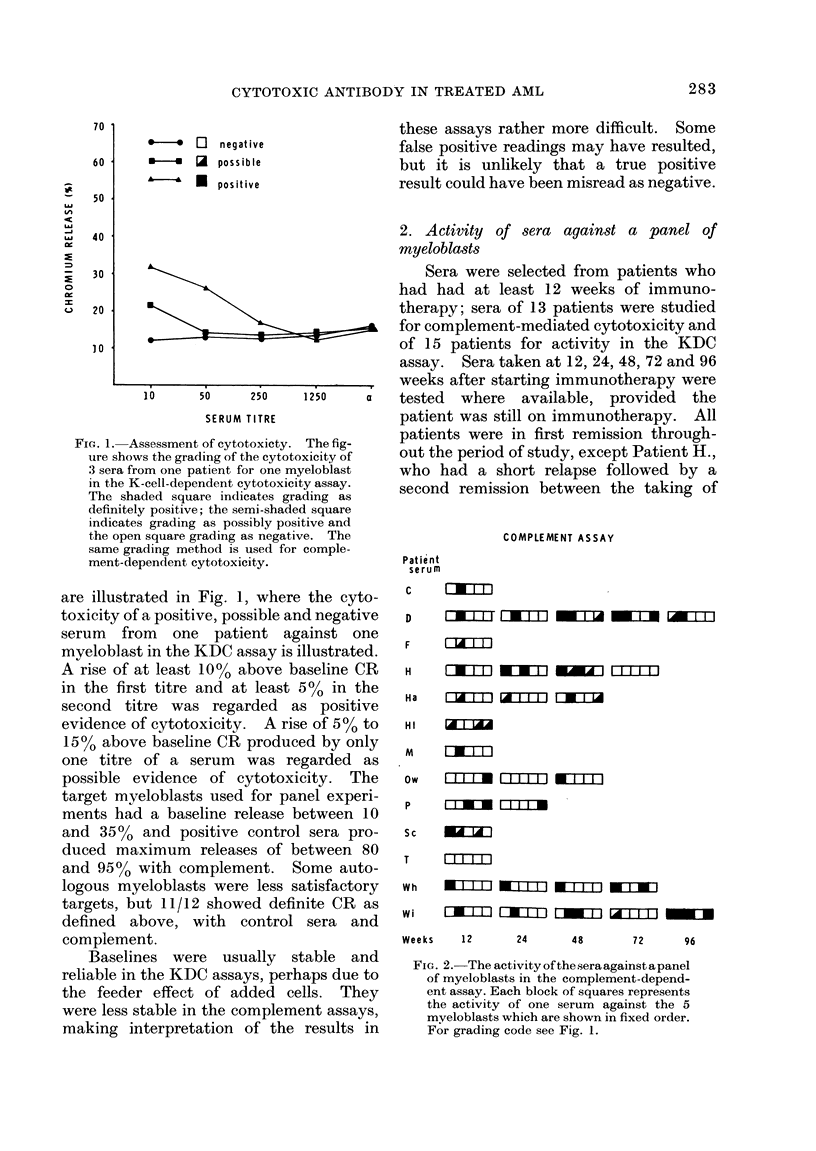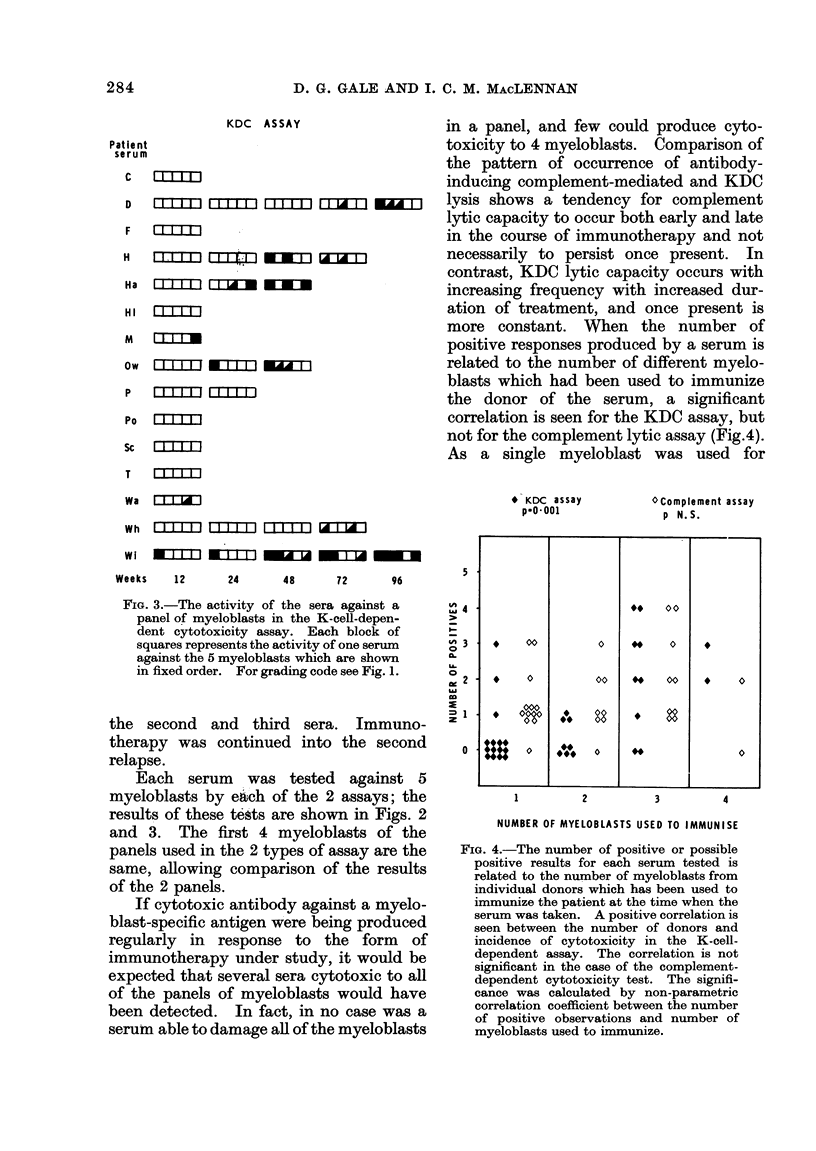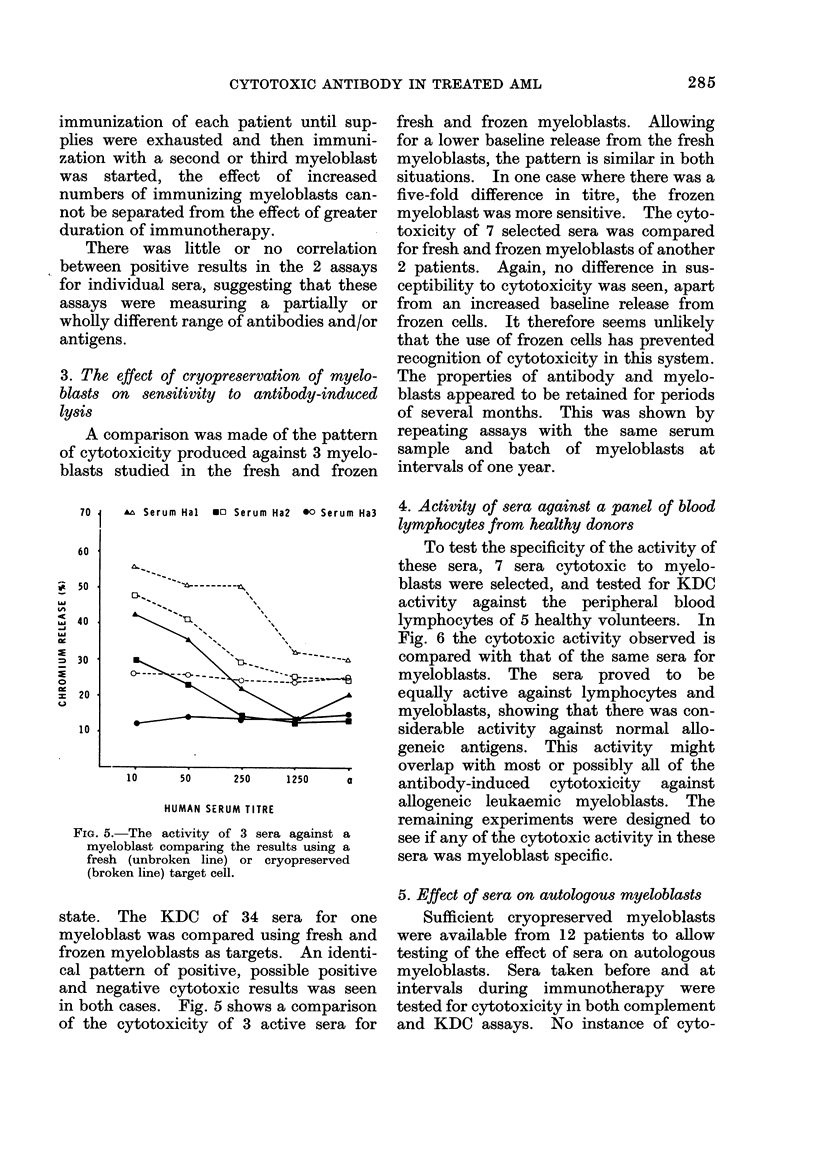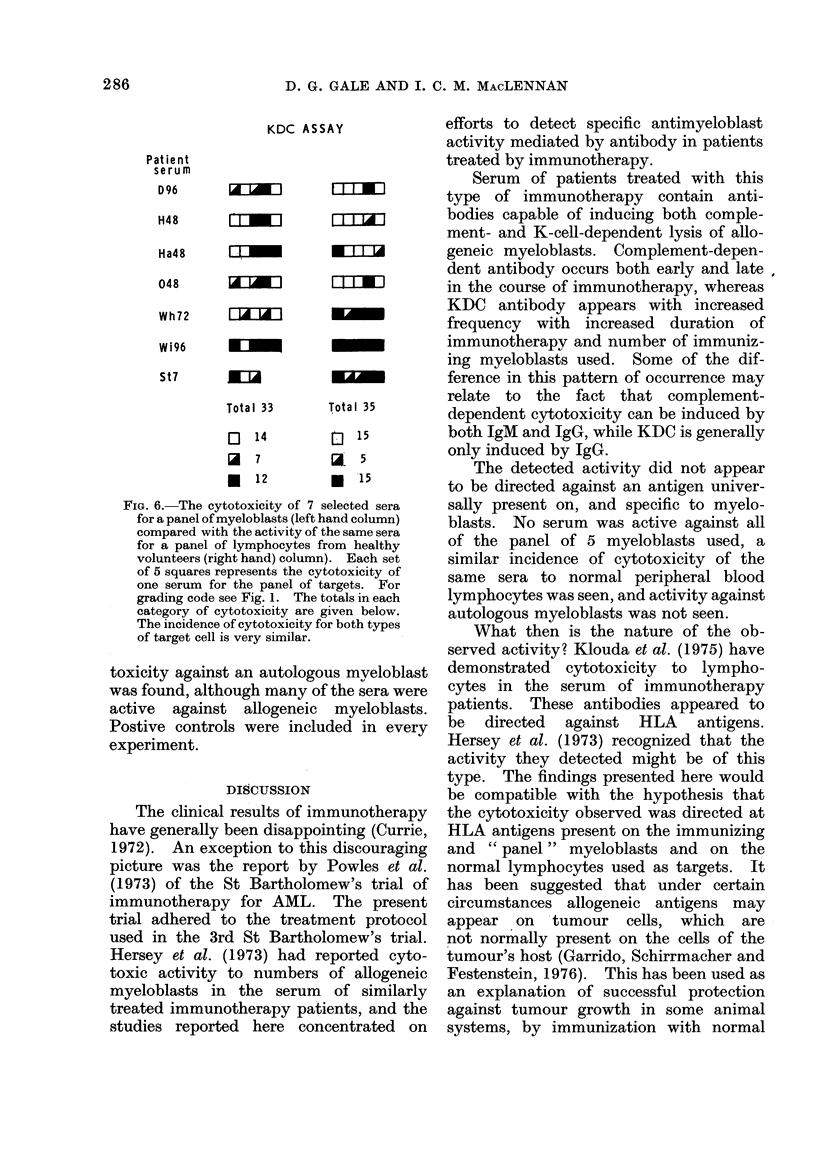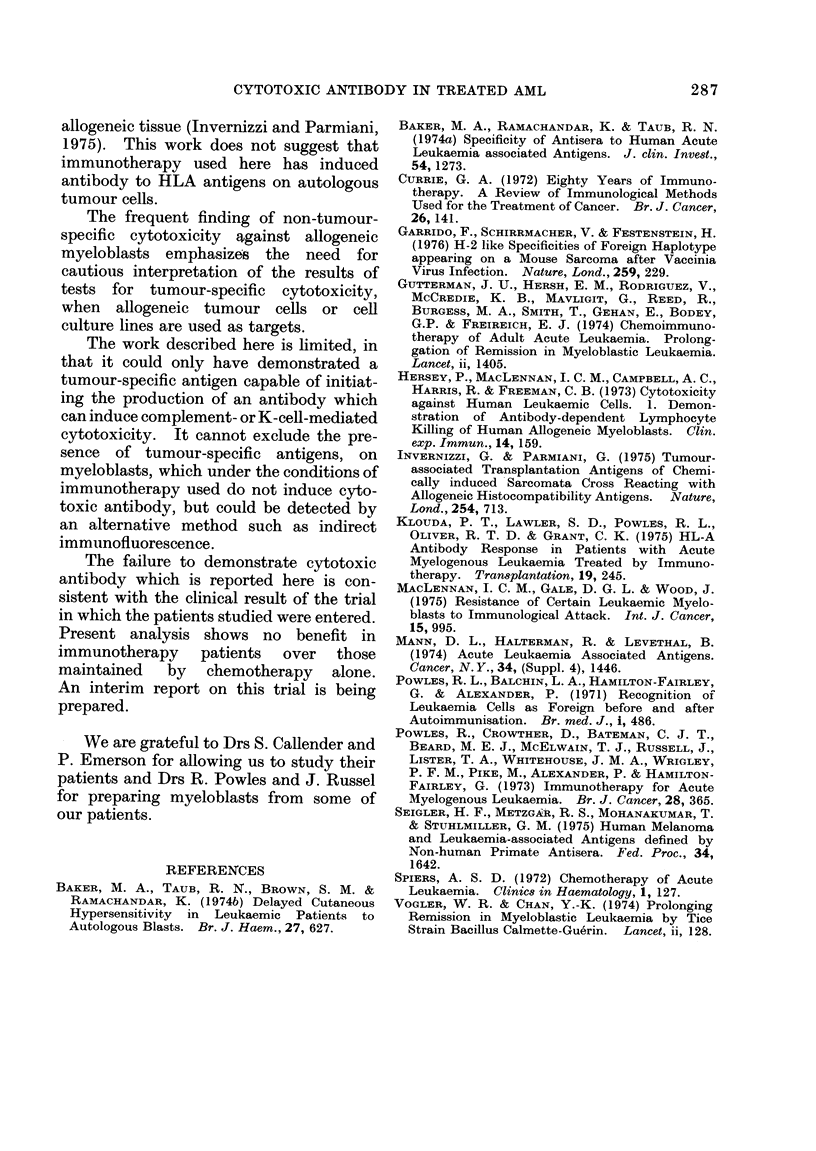# Cytotoxic antibody in acute myeloblastic leukaemia during immunotherapy: lack of tumour specificity.

**DOI:** 10.1038/bjc.1977.40

**Published:** 1977-03

**Authors:** D. G. Gale, I. C. MacLennan

## Abstract

Cytotoxic antibodies to antigens specific for leukaemic myeloblasts have been sought in the serum of patients with acute myeloblastic leukaemia treated by immunotherapy with irradiated allogeneic myeloblasts and BCG. Assays of complement- and K-cell-mediated activity were used. Cytotoxicity to allogeneic myeloblasts was detected in both assays. When sera from 15 patients, taken at various times during immunotherapy, were systematically tested against a panel of 5 myeloblasts, the following patterns emerged: 1. No antibody was cytotoxic against all myeloblasts of the panel in either the K-cell or complement-dependent assay. However, all myeloblasts of the panel were lysed by a number of sera. 2. Cytotoxic antibody was detected as often against a panel of lymphocytes from healthy donors as against the panel of allogeneic myeloblasts. 3. Fresh and cryopreserved myeloblasts were equally susceptible to lysis in both assays. 4. Experiments failed to demonstrate any deterioration of cytotoxic antibody on storage. 5. The number of K-cell-revealed cytotoxic antisera increased with length of immunotherapy. This pattern was not apparent for antibodies revealed by complement. 6. No instance of cytotoxicity in either assay was seen when serum was tested against 12 autologous myeloblasts. It is considered that cytotoxic antibody detected with allogeneic myeloblasts is probably directed against HLA antigens common to immunizing and test target myeloblasts and target lymphocytes.


*Br. J. Cancer* (1977) **35**, 280.

### CYTOTOXIC ANTIBODY IN ACUTE MYELOBLASTIC LEUKAEMIA DURING IMMUNOTHERAPY: LACK OF TUMOUR SPECIFICITY

D. G. GALE AND I. C. M. MacLENNAN

*From the Clinical Immunology Unit, Nuffield Department of Clinical Medicine, Radcliffe Infirmary, Oxford*

Received 20 September 1976 Accepted 18 October 1976

**Summary.—Cytotoxic antibodies to antigens specific for leukaemic myeloblasts have been sought in the serum of patients with acute myeloblastic leukaemia treated by immunotherapy with irradiated allogeneic myeloblasts and BCG. Assays of complement- and K-cell-mediated activity were used. Cytotoxicity to allogeneic myeloblasts was detected in both assays.**

**When sera from 15 patients, taken at various times during immunotherapy, were systematically tested against a panel of 5 myeloblasts, the following patterns emerged:**

**1. No antibody was cytotoxic against all myeloblasts of the panel in either the K-cell or complement-dependent assay. However, all myeloblasts of the panel were lysed by a number of sera.**

**2. Cytotoxic antibody was detected as often against a panel of lymphocytes from healthy donors as against the panel of allogeneic myeloblasts.**

**3. Fresh and cryopreserved myeloblasts were equally susceptible to lysis in both assays.**

**4. Experiments failed to demonstrate any deterioration of cytotoxic antibody on storage.**

**5. The number of K-cell-revealed cytotoxic antisera increased with length of immunotherapy. This pattern was not apparent for antibodies revealed by complement.**

**6. No instance of cytotoxicity in either assay was seen when serum was tested against 12 autologous myeloblasts. It is considered that cytotoxic antibody detected with allogeneic myeloblasts is probably directed against HLA antigens common to immunizing and test target myeloblasts and target lymphocytes.**

CHEMOTHERAPY and supportive treatment in acute myeloblastic leukaemia (AML) has improved to the extent of achieving complete remission in over half the patients in a number of series (Spiers, 1972). However, the length of remissions is disappointingly short and, while half of first remissions may exceed a year, only a handful of patients are alive 3 years after presentation. This situation has provided a strong impetus to find improved methods of sustaining remission in this disease. Immunotherapy is among the approaches tried, and some encouraging results have been reported (Powles *et al.*, 1973; Gutterman *et al.*, 1974; Vogler and Chan, 1974). The data from the St Bartholomew's Hospital study (Powles *et al.*, 1973) are of particular interest, for this trial included concurrent controls treated with chemotherapy. The immunotherapy patients received the same chemotherapy, but were also given $10^9$ allogeneic irradiated cryopreserved leukaemic myeloblasts and live BCG weekly after achieving remission.

One of the rationales for including allogeneic myeloblasts in immunotherapy is that these might share antigens with the patients' own leukaemic cells. However,

This work is supported by the Medical Research Council.
Correspondence should be addressed to Dr I. C. M. MacLennan.

it should be stressed that the existence of common tumour-specific antigens in acute myeloblastic leukaemia has not yet been proved, despite considerable work on this topic. Autologous myeloblasts have been shown to produce delayed-hypersensitivity-type skin responses at some time in the course of the disease in most patients (Baker *et al.*, 1974*b*), and can also stimulate blastogenesis of autologous lymphocytes (Powles *et al.*, 1971). Although these data are consistent with the presence on the myeloblast of an antigen foreign to the autologous host, these studies do not demonstrate that the antigens on different myeloblasts are similar.

Various attempts have been made to produce a xenogeneic serum which will recognize myeloblastic-leukaemia-specific antigen. Baker, Ramachandar and Taub (1974*a*) in mice, and Mann, Halterman and Leventhal (1974) in rabbits, have produced sera which they regarded as probably myeloblast-specific. Seigler *et al.* (1975), working with chimpanzees, have not been able to produce sera reacting with all myeloblasts, and suggest that these more closely related animals may be able to recognize differences in the surface structure of human leukaemic myeloblasts to which lower animals are " blind ". They have produced antisera which react with groups of myeloblasts, though not with all. All these studies have used absorption with normal human tissue to produce sera thought to be more reactive against myeloblasts than normal tissues, but critical appraisal of these data leaves some doubt as to whether they conclusively demonstrate tumour-specific antigens.

Hersey *et al.* (1973) previously described a high incidence of antibody which was cytotoxic against allogeneic myeloblasts, in AML patients receiving immunotherapy. These authors pointed out that this activity could have been directed against normal transplantation antigens. This study seeks to assess the specificity of such antibodies in sera from patients undergoing immunotherapy in the 6th Medical Research Council trial of treatment in acute myeloblastic leukaemia.

### PATIENTS

All material studied was from patients in the Medical Research Council's 6th trial of therapy in AML. The treatment protocol was essentially that used in the St Bartholomew's Hospital 3rd trial (Powles *et al.*, 1973). After induction of remission, patients were given one further 5-day course of chemotherapy followed by weekly injections of $10^9$ irradiated allogeneic cryopreserved myeloblasts in divided doses in 3 limbs subcutaneously and intradermally. Live BCG (Glaxo) was inoculated into the fourth limb by Heaf technique. Each limb was used for the BCG administration in rotation. Maintenance chemotherapy was given for 5 days during each 4th week for the first 12 months of remission.

### METHODS

### (i) *Target cells*

The leukaemic myeloblasts used in this study were collected from patients entered in the 6th MRC trial who had high peripheral white cell counts at presentation. In a few cases, the myeloblasts were collected using a continuous-flow cell separator (International Business Machines) at the Royal Marsden Hospital or Manchester Royal Infirmary. The blood was collected into acid citrate dextrose and allowed to sediment at 1 *g* for 1–2 h. The supernatant leucocyte-rich plasma above the red cells was collected and centrifuged. The cell residue was resuspended in autologous plasma or Hepes-buffered Eagle's basal medium (BME) with 10% foetal bovine serum (FBS). After addition of 10% dimethyl sulphoxide the cells were frozen at − 1°C per minute to about − 60°C and then stored over liquid $N_2$.

The myeloblasts used for the major part of this work were chosen from those in store at Oxford, because of their availability in suitable numbers, viability and favourable labelling characteristics. None of the panel myeloblasts were derived from, or had been used for immunization of any of the patients whose sera were tested. Cytocentrifuge preparations were made of aliquots of all samples used, or from samples from the same batch. In all cases, at least 90% of the cells in the preparations were judged on morphological grounds to be myeloblasts: the remainder were lymphocytes. The myeloblasts were thawed rapidly and diluted drop by drop over about 10 min with 10% FBS in saline at 30°C. After centrifugation, approximately 2–3 × $10^7$ myeloblasts were suspended in 0·5 ml BME + FBS with 100 $\mu$Ci $^{51}$Cr-chromate (Radiochemical Centre, Amersham) and placed in a 30-ml universal container. After incubation at 37°C for 2 h, 20 ml BME was added and 1·5 ml FBS carefully layered under the suspension. After centrifugation, the supernatant was removed and the myeloblasts resuspended at a concentration to give approximately 2 × $10^4$ cells per well. In the labelling conditions used by us the incorporation of sodium [$^{51}$Cr] chromate by myeloblasts is somewhat greater than that by lymphocytes (MacLennan, Gale and Wood, 1975). It can therefore be assumed that well over 90% of label in these preparations was in myeloblasts.

Lymphocytes to be used as targets were separated from heparinized blood of normal volunteers by Ficoll–Triosil gradient. After 2 washes in BME the labelling procedure used for myeloblasts was followed.

(ii) *Test sera*

Sera from patients receiving immunotherapy were collected from fresh, defibrinated blood and stored at −12°C until used. Dilutions of 1/5, 1/20, 1/80, 1/320 and 1/1280 were used for complement-induced lysis. Dilutions of 1/10, 1/50, 1/250, 1/1250 and 1/$\alpha$ were used for assays of antibody-induced lymphocyte-dependent cytotoxicity (K-cell-dependent cytotoxicity or KDC). Two rat sera known to have cytotoxic activity against most myeloblasts were included in each assay as a positive control. They were prepared by pooling the sera of 3 rats, bled out 14 days after i.p. injection of 5 × $10^7$ human leukaemic myeloblasts.

(iii) *Cytotoxic mediators*

(a) *Complement*.—Fresh normal human serum was frozen in liquid $N_2$. Aliquots of this were thawed for use as a complement source. More than one pool was used but all had good lytic activity against myeloblasts sensitized with positive control sera. It was used in a final concentration of 20%.

(b) *Lymphocytes* (*K cells*).—For KDC assays, lymphocytes were collected from defibrinated normal human blood by gelatine sedimentation. After centrifugation and one wash in BME, the lymphocytes were resuspended and used at 3·5 × $10^5$/well. Several normal volunteers participated but only one donor was involved in testing each myeloblast against the whole batch of sera.

(iv) *Test procedure*

All experiments were carried out in sterile Biocult 250-$\mu$l round-bottomed-well microplates with 96 wells. All experiments were carried out in duplicate, with a final volume of 200 $\mu$l per well. The basic medium used was BME, supplemented with glutamine, non-essential aminoacids and penicillin and streptomycin. FBS was not added except at the stage of labelling. As far as possible, the medium was used at 37°C at all times.

Labelled target cells, test serum and mediators were mixed in the concentration and volumes indicated above and incubated at 37°C. Complement assays were incubated for $2\frac{1}{2}$ h and KDC assays for 6 h. After incubation the plates were centrifuged at 100 *g* for 5 min and 150 $\mu$l of supernatant was pipetted off and retained for counting. To measure total $^{51}$Cr per well, 4 aliquots of the target-cell mixture containing 2 × $10^4$ cells were retained for separate counting. Gamma emission of the samples was counted, using a Wallac automatic well scintillation counter. The percentage chromium release (CR) was calculated by the formula:

$$CR = \frac{(1{\cdot}33 \times S - \text{background})}{(T - \text{background})} \times 100$$

where

S = ct/s of supernatant.

T = total well ct/s (mean counts of aliquots of myeloblast mixture).

background = background irradiation in ct/s (taken to be a constant, 0·9 ct/s).

### RESULTS

1. *Assessment of cytotoxicity*

Results were graded as being positive, possibly positive or negative for cytotoxicity. The criteria for this assessment

FIG. 1.—Assessment of cytotoxicty. The figure shows the grading of the cytotoxicity of 3 sera from one patient for one myeloblast in the K-cell-dependent cytotoxicity assay. The shaded square indicates grading as definitely positive; the semi-shaded square indicates grading as possibly positive and the open square grading as negative. The same grading method is used for complement-dependent cytotoxicity.

are illustrated in Fig. 1, where the cytotoxicity of a positive, possible and negative serum from one patient against one myeloblast in the KDC assay is illustrated. A rise of at least 10% above baseline CR in the first titre and at least 5% in the second titre was regarded as positive evidence of cytotoxicity. A rise of 5% to 15% above baseline CR produced by only one titre of a serum was regarded as possible evidence of cytotoxicity. The target myeloblasts used for panel experiments had a baseline release between 10 and 35% and positive control sera produced maximum releases of between 80 and 95% with complement. Some autologous myeloblasts were less satisfactory targets, but 11/12 showed definite CR as defined above, with control sera and complement.

Baselines were usually stable and reliable in the KDC assays, perhaps due to the feeder effect of added cells. They were less stable in the complement assays, making interpretation of the results in these assays rather more difficult. Some false positive readings may have resulted, but it is unlikely that a true positive result could have been misread as negative.

### 2. *Activity of sera against a panel of myeloblasts*

Sera were selected from patients who had had at least 12 weeks of immunotherapy; sera of 13 patients were studied for complement-mediated cytotoxicity and of 15 patients for activity in the KDC assay. Sera taken at 12, 24, 48, 72 and 96 weeks after starting immunotherapy were tested where available, provided the patient was still on immunotherapy. All patients were in first remission throughout the period of study, except Patient H., who had a short relapse followed by a second remission between the taking of

FIG. 2.—The activity of the sera against a panel of myeloblasts in the complement-dependent assay. Each block of squares represents the activity of one serum against the 5 myeloblasts which are shown in fixed order. For grading code see Fig. 1.

FIG. 3.—The activity of the sera against a panel of myeloblasts in the K-cell-dependent cytotoxicity assay. Each block of squares represents the activity of one serum against the 5 myeloblasts which are shown in fixed order. For grading code see Fig. 1.

the second and third sera. Immunotherapy was continued into the second relapse.

Each serum was tested against 5 myeloblasts by each of the 2 assays; the results of these tests are shown in Figs. 2 and 3. The first 4 myeloblasts of the panels used in the 2 types of assay are the same, allowing comparison of the results of the 2 panels.

If cytotoxic antibody against a myeloblast-specific antigen were being produced regularly in response to the form of immunotherapy under study, it would be expected that several sera cytotoxic to all of the panels of myeloblasts would have been detected. In fact, in no case was a serum able to damage all of the myeloblasts in a panel, and few could produce cytotoxicity to 4 myeloblasts. Comparison of the pattern of occurrence of antibody-inducing complement-mediated and KDC lysis shows a tendency for complement lytic capacity to occur both early and late in the course of immunotherapy and not necessarily to persist once present. In contrast, KDC lytic capacity occurs with increasing frequency with increased duration of treatment, and once present is more constant. When the number of positive responses produced by a serum is related to the number of different myeloblasts which had been used to immunize the donor of the serum, a significant correlation is seen for the KDC assay, but not for the complement lytic assay (Fig.4). As a single myeloblast was used for

FIG. 4.—The number of positive or possible positive results for each serum tested is related to the number of myeloblasts from individual donors which has been used to immunize the patient at the time when the serum was taken. A positive correlation is seen between the number of donors and incidence of cytotoxicity in the K-cell-dependent assay. The correlation is not significant in the case of the complement-dependent cytotoxicity test. The significance was calculated by non-parametric correlation coefficient between the number of positive observations and number of myeloblasts used to immunize.

immunization of each patient until supplies were exhausted and then immunization with a second or third myeloblast was started, the effect of increased numbers of immunizing myeloblasts cannot be separated from the effect of greater duration of immunotherapy.

There was little or no correlation between positive results in the 2 assays for individual sera, suggesting that these assays were measuring a partially or wholly different range of antibodies and/or antigens.

3. *The effect of cryopreservation of myeloblasts on sensitivity to antibody-induced lysis*

A comparison was made of the pattern of cytotoxicity produced against 3 myeloblasts studied in the fresh and frozen state. The KDC of 34 sera for one myeloblast was compared using fresh and frozen myeloblasts as targets. An identical pattern of positive, possible positive and negative cytotoxic results was seen in both cases. Fig. 5 shows a comparison of the cytotoxicity of 3 active sera for fresh and frozen myeloblasts. Allowing for a lower baseline release from the fresh myeloblasts, the pattern is similar in both situations. In one case where there was a five-fold difference in titre, the frozen myeloblast was more sensitive. The cytotoxicity of 7 selected sera was compared for fresh and frozen myeloblasts of another 2 patients. Again, no difference in susceptibility to cytotoxicity was seen, apart from an increased baseline release from frozen cells. It therefore seems unlikely that the use of frozen cells has prevented recognition of cytotoxicity in this system. The properties of antibody and myeloblasts appeared to be retained for periods of several months. This was shown by repeating assays with the same serum sample and batch of myeloblasts at intervals of one year.

FIG. 5.—The activity of 3 sera against a myeloblast comparing the results using a fresh (unbroken line) or cryopreserved (broken line) target cell.

4. *Activity of sera against a panel of blood lymphocytes from healthy donors*

To test the specificity of the activity of these sera, 7 sera cytotoxic to myeloblasts were selected, and tested for KDC activity against the peripheral blood lymphocytes of 5 healthy volunteers. In Fig. 6 the cytotoxic activity observed is compared with that of the same sera for myeloblasts. The sera proved to be equally active against lymphocytes and myeloblasts, showing that there was considerable activity against normal allogeneic antigens. This activity might overlap with most or possibly all of the antibody-induced cytotoxicity against allogeneic leukaemic myeloblasts. The remaining experiments were designed to see if any of the cytotoxic activity in these sera was myeloblast specific.

5. *Effect of sera on autologous myeloblasts*

Sufficient cryopreserved myeloblasts were available from 12 patients to allow testing of the effect of sera on autologous myeloblasts. Sera taken before and at intervals during immunotherapy were tested for cytotoxicity in both complement and KDC assays. No instance of cyto-

FIG. 6.—The cytotoxicity of 7 selected sera for a panel of myeloblasts (left hand column) compared with the activity of the same sera for a panel of lymphocytes from healthy volunteers (right hand) column). Each set of 5 squares represents the cytotoxicity of one serum for the panel of targets. For grading code see Fig. 1. The totals in each category of cytotoxicity are given below. The incidence of cytotoxicity for both types of target cell is very similar.

toxicity against an autologous myeloblast was found, although many of the sera were active against allogeneic myeloblasts. Postive controls were included in every experiment.

### DISCUSSION

The clinical results of immunotherapy have generally been disappointing (Currie, 1972). An exception to this discouraging picture was the report by Powles *et al.* (1973) of the St Bartholomew's trial of immunotherapy for AML. The present trial adhered to the treatment protocol used in the 3rd St Bartholomew's trial. Hersey *et al.* (1973) had reported cytotoxic activity to numbers of allogeneic myeloblasts in the serum of similarly treated immunotherapy patients, and the studies reported here concentrated on efforts to detect specific antimyeloblast activity mediated by antibody in patients treated by immunotherapy.

Serum of patients treated with this type of immunotherapy contain antibodies capable of inducing both complement- and K-cell-dependent lysis of allogeneic myeloblasts. Complement-dependent antibody occurs both early and late in the course of immunotherapy, whereas KDC antibody appears with increased frequency with increased duration of immunotherapy and number of immunizing myeloblasts used. Some of the difference in this pattern of occurrence may relate to the fact that complement-dependent cytotoxicity can be induced by both IgM and IgG, while KDC is generally only induced by IgG.

The detected activity did not appear to be directed against an antigen universally present on, and specific to myeloblasts. No serum was active against all of the panel of 5 myeloblasts used, a similar incidence of cytotoxicity of the same sera to normal peripheral blood lymphocytes was seen, and activity against autologous myeloblasts was not seen.

What then is the nature of the observed activity? Klouda *et al.* (1975) have demonstrated cytotoxicity to lymphocytes in the serum of immunotherapy patients. These antibodies appeared to be directed against HLA antigens. Hersey *et al.* (1973) recognized that the activity they detected might be of this type. The findings presented here would be compatible with the hypothesis that the cytotoxicity observed was directed at HLA antigens present on the immunizing and " panel " myeloblasts and on the normal lymphocytes used as targets. It has been suggested that under certain circumstances allogeneic antigens may appear on tumour cells, which are not normally present on the cells of the tumour's host (Garrido, Schirrmacher and Festenstein, 1976). This has been used as an explanation of successful protection against tumour growth in some animal systems, by immunization with normal allogeneic tissue (Invernizzi and Parmiani, 1975). This work does not suggest that immunotherapy used here has induced antibody to HLA antigens on autologous tumour cells.

The frequent finding of non-tumour-specific cytotoxicity against allogeneic myeloblasts emphasizes the need for cautious interpretation of the results of tests for tumour-specific cytotoxicity, when allogeneic tumour cells or cell culture lines are used as targets.

The work described here is limited, in that it could only have demonstrated a tumour-specific antigen capable of initiating the production of an antibody which can induce complement- or K-cell-mediated cytotoxicity. It cannot exclude the presence of tumour-specific antigens, on myeloblasts, which under the conditions of immunotherapy used do not induce cytotoxic antibody, but could be detected by an alternative method such as indirect immunofluorescence.

The failure to demonstrate cytotoxic antibody which is reported here is consistent with the clinical result of the trial in which the patients studied were entered. Present analysis shows no benefit in immunotherapy patients over those maintained by chemotherapy alone. An interim report on this trial is being prepared.

We are grateful to Drs S. Callender and P. Emerson for allowing us to study their patients and Drs R. Powles and J. Russel for preparing myeloblasts from some of our patients.

### REFERENCES

BAKER, M. A., TAUB, R. N., BROWN, S. M. & RAMACHANDAR, K. (1974*b*) Delayed Cutaneous Hypersensitivity in Leukaemic Patients to Autologous Blasts. *Br. J. Haem.*, **27**, 627.

BAKER, M. A., RAMACHANDAR, K. & TAUB, R. N. (1974*a*) Specificity of Antisera to Human Acute Leukaemia associated Antigens. *J. clin. Invest.*, **54**, 1273.

CURRIE, G. A. (1972) Eighty Years of Immunotherapy. A Review of Immunological Methods Used for the Treatment of Cancer. *Br. J. Cancer*, **26**, 141.

GARRIDO, F., SCHIRRMACHER, V. & FESTENSTEIN, H. (1976) H-2 like Specificities of Foreign Haplotype appearing on a Mouse Sarcoma after Vaccinia Virus Infection. *Nature, Lond.*, **259**, 229.

GUTTERMAN, J. U., HERSH, E. M., RODRIGUEZ, V., MCCREDIE, K. B., MAVLIGIT, G., REED, R., BURGESS, M. A., SMITH, T., GEHAN, E., BODEY, G.P. & FREIREICH, E. J. (1974) Chemoimmunotherapy of Adult Acute Leukaemia. Prolonggation of Remission in Myeloblastic Leukaemia. *Lancet*, ii, 1405.

HERSEY, P., MACLENNAN, I. C. M., CAMPBELL, A. C., HARRIS, R. & FREEMAN, C. B. (1973) Cytotoxicity against Human Leukaemic Cells. I. Demonstration of Antibody-dependent Lymphocyte Killing of Human Allogeneic Myeloblasts. *Clin. exp. Immun.*, **14**, 159.

INVERNIZZI, G. & PARMIANI, G. (1975) Tumour-associated Transplantation Antigens of Chemically induced Sarcomata Cross Reacting with Allogeneic Histocompatibility Antigens. *Nature, Lond.*, **254**, 713.

KLOUDA, P. T., LAWLER, S. D., POWLES, R. L., OLIVER, R. T. D. & GRANT, C. K. (1975) HL-A Antibody Response in Patients with Acute Myelogenous Leukaemia Treated by Immunotherapy. *Transplantation*, **19**, 245.

MACLENNAN, I. C. M., GALE, D. G. L. & WOOD, J. (1975) Resistance of Certain Leukaemic Myeloblasts to Immunological Attack. *Int. J. Cancer*, **15**, 995.

MANN, D. L., HALTERMAN, R. & LEVETHAL, B. (1974) Acute Leukaemia Associated Antigens. *Cancer, N.Y.*, **34**, (Suppl. 4), 1446.

POWLES, R. L., BALCHIN, L. A., HAMILTON-FAIRLEY, G. & ALEXANDER, P. (1971) Recognition of Leukaemia Cells as Foreign before and after Autoimmunisation. *Br. med. J.*, **i**, 486.

POWLES, R., CROWTHER, D., BATEMAN, C. J. T., BEARD, M. E. J., MCELWAIN, T. J., RUSSELL, J., LISTER, T. A., WHITEHOUSE, J. M. A., WRIGLEY, P. F. M., PIKE, M., ALEXANDER, P. & HAMILTON-FAIRLEY, G. (1973) Immunotherapy for Acute Myelogenous Leukaemia. *Br. J. Cancer*, **28**, 365.

SEIGLER, H. F., METZGAR, R. S., MOHANAKUMAR, T. & STUHLMILLER, G. M. (1975) Human Melanoma and Leukaemia-associated Antigens defined by Non-human Primate Antisera. *Fed. Proc.*, **34**, 1642.

SPIERS, A. S. D. (1972) Chemotherapy of Acute Leukaemia. *Clinics in Haematology*, **1**, 127.

VOGLER, W. R. & CHAN, Y.-K. (1974) Prolonging Remission in Myeloblastic Leukaemia by Tice Strain Bacillus Calmette-Guérin. *Lancet*, ii, 128.